# Internal Medication in Vesical Catarrh

**Published:** 1905-12

**Authors:** 


### ABSTRACTS.

### Internal Medication in Vesical Catarrh.

Prof. C. Posner, (*Berliner kl. Wochenschr.*, January 9, 1905) says that despite the development of local measures, the internal indications in vesical catarrh are most important. The practitioner is naturally desirous of attaining his ends by internal medication alone; almost all acute cases respond best to the latter; and many a chronic case is much better off if the urinary passages are left alone. Even where instrumentation is necessary, medicinal treatment is indispensable.

Faith in mineral waters has, however, lost ground; and since Nicolaier introduced urotropin, the question of medication seemed essentially settled. We regard every vesical catarrh as the result of an infection; pathogenic germs have reached the bladder and set up a cystitis which can only be combated by destroying the microbes or hindering their growth. Urotropin seemed especially suited for the purpose, since it liberates in the body the very efficient disinfectant, formaldehyd.

Experience confirmed these expectations, often in a surprising manner. From all sides there came confirmations of the claims Nicolaier made in 1895. The curative effects of urotropin in the cystites of adults and children was abundantly demonstrated; the cloudy urines cleared, often with marvelous rapidity; and the continuous use of the drug had no injurious effect. Thus, Heubner gave it for very prolonged periods in the obstinate cystites of children. Urotropin was also found to be an effective preventive of catheter infection. Most physicians take advantage of this when they begin treatment of prostatic hypertrophy. It shows similar prophylactic properties in general infections; thus in typhoid fever it protects the urinary organs from the injurious action of the excreated bacilli; and even in scarlatina it prevents the dreaded nephritis. There are but few remedies concerning which theory and practice are so thoroughly in accord.

Of course there are occasional cases in which it does not act, and the idea has arisen that it is still not active enough as a disinfectant and that by changes of chemical constitution and mixtures, beter results could be obtained. Such assumptions are erroneous. The variability in its action is, in my opinion, due to qualitative and not to quantitative differences in the infective agent. Certain cystites have been found by the earliest observers to be but little amenable to urptropin; in tubercular and gonorrheal cystitis the drug only combats mixed infection, if present. The domain of urotropin is the ordinary infections with coli bacilli, staphylococci, etc., as in catheter infection, urethral strictures, prostatic hypertrophy, etc. In these, no matter whether mild or severe, whether urine is acid or alkiline, urotropin is the most valuable remedy we possess. In fact, when urotropin is entirely ineffective in an apparently benign chronic cystitis, it should arouse suspicion of tuberculosis, exactly as when the local use of silver nitrate does harm instead of good.

In the endeavor to enhance the action of urotropin, a combination with methylene-citric-acid has been produced under the names of helmitol and new-urotropin. Very soon after their introduction I experimented with both in my private practice and in the polyclinic; my results will be reported elsewhere by Dr. J. Vogel. I aimed at determining whether the new remedy was effective in cases where urotropin failed; but found that in these cases it gave no results at all; both to us and to the patients it seemed absolutely the same whether urotropin or the new compound was given. Other observers, such as Goldberg, obtained the same results. Nicolaier recently showed that methylene-citric-acid itself has extremely slight disinfectant power in the urine, so that urotropin, of which helmitol and new-urotropin contain only 40.7%, is the effective agent in the combination. This is shown by the fact that the latter must be given in double the dose of urotropin, and hence is twice as expensive in use. Besides, Goldberg reports a remarkably large proportion of injurious by-effects, especially hematuria, after the use of helmitol.

Hence helmitol cannot be regarded as a theraputic advance. We had no better results with other recently introduced compounds. Thus hetralin proved in our experience to be in no way better than urotropin. As an internal disinfectant, especially in cystitis, due to coli bacilli and staphylococci, no drug can replace urotropin.

